# Investigating gene-microRNA networks in atrial fibrillation patients with mitral valve regurgitation

**DOI:** 10.1371/journal.pone.0232719

**Published:** 2020-05-11

**Authors:** Joana Larupa Santos, Ismael Rodríguez, Morten S. Olesen, Bo Hjorth Bentzen, Nicole Schmitt

**Affiliations:** 1 Department of Biomedical Sciences, Faculty of Health and Medical Sciences, University of Copenhagen, Copenhagen N, Denmark; 2 Department of Cardiology, Laboratory for Molecular Cardiology, The Heart Centre, Rigshospitalet, University Hospital of Copenhagen, Copenhagen Ø, Denmark; Kunming University of Science and Technology, CHINA

## Abstract

**Background:**

Atrial fibrillation (AF) is predicted to affect around 17.9 million individuals in Europe by 2060. The disease is associated with severe electrical and structural remodelling of the heart, and increased the risk of stroke and heart failure. In order to improve treatment and find new drug targets, the field needs to better comprehend the exact molecular mechanisms in these remodelling processes.

**Objectives:**

This study aims to identify gene and miRNA networks involved in the remodelling of AF hearts in AF patients with mitral valve regurgitation (MVR).

**Methods:**

Total RNA was extracted from right atrial biopsies from patients undergoing surgery for mitral valve replacement or repair with AF and without history of AF to test for differentially expressed genes and miRNAs using RNA-sequencing and miRNA microarray. *In silico* predictions were used to construct a mRNA-miRNA network including differentially expressed mRNAs and miRNAs. Gene and chromosome enrichment analysis were used to identify molecular pathways and high-density AF loci.

**Results:**

We found 644 genes and 43 miRNAs differentially expressed in AF patients compared to controls. From these lists, we identified 905 pairs of putative miRNA-mRNA interactions, including 37 miRNAs and 295 genes. Of particular note, AF-associated miR-130b-3p, miR-338-5p and miR-208a-3p were differentially expressed in our AF tissue samples. These miRNAs are predicted regulators of several differentially expressed genes associated with cardiac conduction and fibrosis. We identified two high-density AF loci in chromosomes 14q11.2 and 6p21.3.

**Conclusions:**

AF in MVR patients is associated with down-regulation of ion channel genes and up-regulation of extracellular matrix genes. Other AF related genes are dysregulated and several are predicted to be targeted by miRNAs. Our novel miRNA-mRNA regulatory network provides new insights into the mechanisms of AF.

## Introduction

Atrial Fibrillation (AF) is the most common type of cardiac arrhythmia. The disease represents a major economic burden and prevalence is predicted to increase over the next decades both in Europe and the United States [1]. Genetic variants are well documented in cases of early-onset and ‘lone’ AF [2,3]. Additionally, more than one hundred susceptibility loci were identified by population-based, genome-wide association studies (GWAS) [4,5].

The vast majority of AF patients are over 75 years old indicating that AF is an age-related disease. The disease often starts by short self-terminating episodes originating from a trigger in a vulnerable substrate that leads to rapid focal ectopic firing and re-entry of electrical signals in the atria. In some cases, AF can gradually develop to a permanent condition through a process called remodelling [6]. Electrical remodelling results from ion channel dysregulation while structural remodelling is characterized by increased fibrosis, atrial dilation and conduction abnormalities [7,8]. The available therapeutic options in clinical practice have limited efficiency, especially in those patients with more progressed AF, likely due to higher severity of tissue remodelling [9].

Valvular heart disease (VHD) is an established risk factor for AF [10]. However, not all patients with valve disorders develop AF, suggesting that some VHD patients are more predisposed to develop AF. Differences in the remodelling of affected hearts might be a secondary factor predisposing to AF in these patients.

MicroRNAs (miRNAs) are a class of highly conserved short non-protein-coding RNAs. These 18–24 nucleotides RNA molecules are known to negatively regulate the expression of complementary target genes by binding to the 3’-untranslated region (UTR) of messenger RNAs (mRNAs) in the cytosol, promoting either mRNA degradation or inhibiting translation [11]. MiRNAs can be classified as intergenic, intronic and exonic, and the expression of intronic and exonic miRNAs is mainly controlled by the promoter of the host gene [12].

Original reports on the role of miR-1 in cardiogenesis have triggered a large body of research dealing with the role of miRNAs in cardiac development and pathological processes [13]. Following this report, abnormal expression of many other miRNAs has been described in various types of cardiac disorders [14]. In the context of AF, different studies have shown a role of miRNAs in the deregulation of ion channels based on gene expression analyses of tissue samples from AF patients and additional functional studies [15–17]. Cardiac fibrosis was also shown to be influenced by a number of miRNAs, such as miR-133a [18,19]. However, much is yet to be learned about the complete role of miRNAs in arrhythmogenesis. Hence, the analysis of miRNAs expression can give important insight into regulatory mechanisms involved in disease, especially when combined with gene expression profiling studies [20,21].

The present study identifies several miRNAs and genes that may contribute to the development of AF-associated with VHD and illustrates a miRNA-mRNA network with hundreds of putative regulatory interactions from studying tissue samples from AF patients and controls with mitral valve regurgitation (MVR).

## Materials and methods

### Patients and tissue samples

Right atrial (RA) posterior wall tissue biopsies were obtained from unrelated Caucasian patients undergoing open-heart surgery for mitral valve replacement or repair at the University Hospital of Copenhagen (Rigshospitalet), as previously described [22]. From these, six patients with AF sustained for at least two months were selected. The control group included six patients in sinus rhythm (SR) and with no previous history of AF who were selected according to age and gender match. Cardiac disease, hypertension and type 2 diabetes mellitus were used as exclusion criteria.

The study was approved by the Ethics Committee of the Capital Region of Copenhagen (protocol reference no. 16238), in accordance with the Declaration of Helsinki. Written informed consent for the use of clinical information and biological samples was provided by all participants. Clinical data from all subjects was obtained through questionnaires and clinical records from the Danish health care system.

### RNA preparation

Total RNA including small RNAs was extracted from RA biopsies of six AF patients and six control patients using the miRNeasy kit (QIAGEN, Hilden, Germany) according to manufacturer’s instructions. RNA concentration was measured in a NanoDrop 2000 (ThermoScientific, Wilmington, USA) and quality was assessed in a 2100 Bioanalyzer (Agilent Technologies, Santa Clara, CA, USA). RNA samples with a RIN > 6 were used in further experiments. For details see Supplementary methods in S1 File.

### RNA-sequencing

Libraries were prepared from ribosomal RNA depleted RNA samples using the TruSeq Stranded Total RNA Library Prep Kit (Illumina, San Diego, California, USA). Libraries were sequenced on an Illumina HiSeq 2500. The control sample HS24 was not used due to low amount of input RNA. The raw sequencing data is available in the Electronic Research Data Archive (University of Copenhagen), see Data Availability section.

### Transcriptome sequencing analysis

Raw paired-end reads were aligned to the human GRCh38 reference transcriptome using the Kallisto pseudoaligner with default options [23]. Kallisto quantifies the abundance of reads on transcript level. The resulting quantification files were imported to R for downstream analysis. Transcript counts were collapsed to gene level. Differentially expressed genes (DEGs) in AF compared to controls were obtained using the Bioconductor package DEseq2 [24]. Genes with false discovery rate < 0.05 and log2fold-change (FC) above 1 or below -1 were considered DEGs. Principal component analysis (PCA) and unsupervised hierarchical clustering of sample-to-sample distance matrixes were used to analyse sample clustering according to transcriptomic similarities.

DEGs were compared with genes of interest including 1) AF-associated genes from genomic, transcriptomic and proteomic studies from a broad literature search and 2) genes involved in mechanisms of interest, such as fibrosis, cardiac contraction and ion channel currents, from GO terms and HUGO Gene Nomenclature Committee.

### MiRNAs microarray

Microarrays were performed with the exact same RNA samples used for the RNA-seq experiments. Mature miRNA transcripts were hybridized to the GeneChipS1 miRNA 4.0 Array (Affymetrix, Santa Clara, USA). Raw CEL data files are available at the Electronic Research Data Archive (University of Copenhagen), see Data Availability section.

Data generated by the Affymetrix’s miRNA array was normalized using the robust multi-array average method [25]. Quality metrics showed one control sample (HS24) as an outlier (S1 Fig in S1 File). FC values and p-values (*p*) of expression changes were calculated using the Limma package in R/Bioconductor project [26]. A cut-off *p* < 0.01 was used for selection of differentially expressed miRNAs. No FC cut-off was applied. Samples and differentially expressed miRNAs were subjected to unsupervised hierarchical clustering and plotted as a heat map.

### Validation of RNA-seq and microarray by quantitative PCR

Qualitative polymerase chain reaction (qPCR) was performed to confirm the reliability of RNA-seq and microarray data. cDNA was synthesized using the Precision nanoScript2 Reverse Transcription kit (PrimerDesign, Southampton, United Kingdom) for mRNAs and the Universal cDNA Synthesis kit II (Exiqon, Wobrun, Massachusetts, USA) for miRNAs. The expression of selected genes was measured using Taqman double dye probes and PrecisionPLUS MasterMix with ROX (PrimerDesign, Southampton, United Kingdom). The miRNAs qPCR reactions were performed using commercial miRCURY LNA^™^ Universal RT microRNA PCR primers and the ExiLENT SYBR® Green master mix (Exiqon, Wobrun, Massachusetts, USA). All experiments were performed in a light cycler CFX Connect Real-Time System (BIO-RAD, Hertfordshire, UK). The following genes–*KCNA4*, *ACTN2*, *KCNK3*, *NANOG*, *TNNT2*, *KCNQ5*, *KCNJ5* and *KCNB1*, and miRNAs—miR-143-5p, miR-192-5p, miR-187-3p, miR-208b-3p, miR-338-5p, miR-335-5p, miR-432-5p, miR-490-5p, miR-499a-5p, miR-503-5p and miR-92b-3p, were investigated. *YWHAZ* and RPL13A were used as references to normalize the results, while miRNA-16-5p and miR-103a-3p were used to normalize the miRNAs results. Relative expression and FC values were calculated using the 2^-ΔΔCt^ method.

### Statistical analysis

Statistical analysis was performed on GraphPad Prism 7 (GraphPad Software Inc., San Diego, USA). Mann-Whitney U-test and was applied for comparison of continuous variables. Patient data is presented as mean ± standard deviation (SD) and percentage. *P* < 0.05 was considered statistically significant.

### miRNA target genes prediction

Candidate target genes of the differentially expressed miRNAs were obtained using the miRWalk3 target prediction database [27,28]. The programme output includes predictions from miRWalk’s machine learning approach (TarPmiR) and experimentally validated interaction from miRTarBase. The 3’-UTR of genes was selected as putative binding site and only predictions with a binding probability above 0.95 were accepted. Afterwards, miRNA-gene interactions in which the target gene was included in our DEGs list were kept and all the others were filtered out. Normalized expression levels from microarray and RNA-seq were used to evaluate the correlation between each miRNA-gene pair using Spearman’s correlation coefficient in order to find monotonic relations, rather than just linear, given the different magnitudes used for the RNA-seq and microarray experiments. Subsequently, interaction with a negative correlation below -0.5 were used to construct a miRNA-gene regulatory network in Cytoscape version 3.6.1 [29]. Filtering criteria was applied to the networks in order to help visualize and identify relevant interactions. Secondary miRNA-gene networks were built for up- and down-regulated miRNAs previously association with AF in expression studies [30–32] and respective predicted target genes.

### Functional enrichment analysis

To better understand the functional role of differentially expressed miRNAs and target genes, a functional enrichment analysis of the targeted genes after filtering was performed using the Database for Annotation, Visualization and Integrated Discovery (DAVID) [33]. The same analysis was performed with the full list of DEGs from RNA-seq experiments. Our enrichment analysis was focused on gene ontology (GO) terms and Kyoto Encyclopedia of Genes and Genomes (KEGG) pathways using an enrichment cut-off of *p* < 0.05 after Benjamini-Hochberg correction.

### Positional enrichment of genetic elements

Up- and down-regulated genes and miRNAs were plotted together with AF-associated single nucleotide polymorphisms (SNPs), according to chromosome position, to visualize possible clusters of these genetic elements. AF SNPs were retrieved from published data and the Human Short Variants dataset from Ensembl Variation 96 [34]. The location of the published SNPs had to be converted from Hg19 assembly to Hg38 using the UCSC liftOver tool. For the rest of the elements, genomic location and cytogenetic band information was retrieved from Biomart (Bioconductor). The nearest gene(s) to each SNP genome coordinate were obtained to plot together with the matching SNP. For 81 SNPs retrieved, no associated gene was found. A cluster was defined as two or more consecutive elements overlapping or not further than 20 kbp. Clusters including five or more co-localized elements of any type were considered as high-density regions. Subsequently, high-density regions comprising exclusively SNPs were excluded from our analysis. The total number of elements per chromosome was normalized to chromosome size and/or gene density. Lastly, the genomic location of DE miRNAs and genes was used to assess co-expression patterns between intronic or exonic miRNAs and host genes.

## Results

### Patient characteristics

RA posterior wall biopsies were obtained from MVR patients with (n = 6) and without (n = 6) AF undergoing surgery for valve replacement or repair. Clinical characteristics of all subjects are listed in Table 1. There were no significant differences between the groups apart from AF history. Left atrial (LA) size was increased in both groups. Ejection fraction (EF) was lower in AF subjects but the difference was not significant. EF values were unavailable in one subject from each group, while one LA size measurement was unavailable in the AF group.

**Table 1:** Clinical characteristics of the study population.

	AF patients (N = 6)	Controls (N = 6)
Gender	3 males (50)	3 males (50)
Age (years)	59.3 ± 9.2	59.3 ± 8.7
BMI (kg/m^2^)	24.6 ± 1.6	22.5 ± 3.3
AF Type: Paroxysmal	0 (0)	NA
	2 (33.3)	NA
	4 (66.7)	NA
AF duration (months)	2–14	NA
Smoking	3 (50)	2 (33.3)
Alcohol consumption (units/week)		
≤7	4 (66.7)	2 (33.3)
8–14	1 (16.7)	2 (33.3)
15–21	1 (16.7)	2 (33.3)
>21	0 (0)	0 (0)
Hypertension	0 (0)	0 (0)
Type II diabetes mellitus	0 (0)	0 (0)
Heart failure symptoms (NYHA):		
I	1 (16.7)	1 (16.7)
II	3 (50)	4 (66.7)
III	2 (33.3)	1 (16.7)
IV	0 (0)	0 (0)
Left atrial dilation^1^^,^^2^ [35]:		
Normal	0 (0)	0 (0)
Moderate	1 (16.7)	1 (16.7)
Severe	4 (66.7)	5 (83.3)
Ejection Fraction^1^	56 ± 6.5	67 ± 9.7

^1^ One or two unavailable values

^2^ Determined using biplane Simpson model; AF = atrial fibrillation; BMI = body mass index; NA–not applicable; NYHA = New York Heart Association classification; Values are shown as n (%) or mean ± SD.

### Gene expression signature of AF tissues

**Fig 1:**
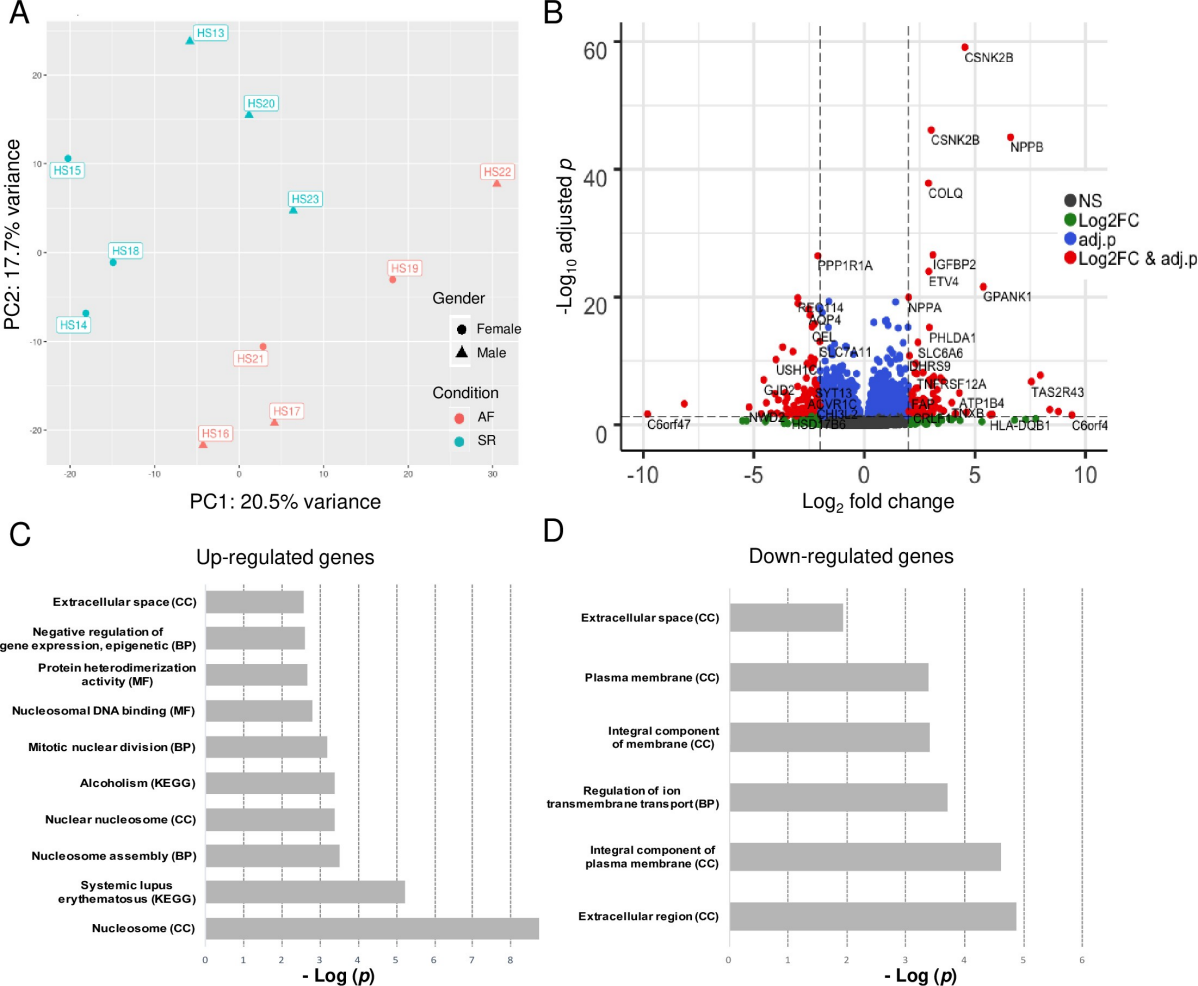
Transcriptome analysis of right atrial biopsies from AF patients compared to controls (SR). A. Principal component analysis (PCA) showing the overall effect of variances between the transcriptome of samples analysed by RNA-sequencing. B. Volcano plot comparing expression of 44,852 genes in the right atrium of AF patients in relation to SR. Red dots represent genes with adjusted-*p* < 0.05 and log_2_FC > 2 or < -2. C-D. Gene set enrichment analysis of differentially expressed genes. Top 10 enriched gene ontology terms and pathways with an enrichment *p*-value < 0.05 after Benjamini-Hochberg correction from up- (C) and down-regulated (D) genes were plotted. AF–atrial fibrillation; BP–biological processes; CC–cellular component; KEGG–Kyoto Encyclopedia of Genes and Genomes; MF–molecular function; NS–non-significant; FC—fold change.

We investigated differences in gene expression between AF and control patients using RNA-sequencing. PCA showed that males and females in the AF group were separated by PC1 (Fig 1A). No other characteristics reported in Table 1 contribute to the clustering of samples. S2 Fig in S1 File represents clustering of samples according to sample-to-sample matrix distances. From the 44,852 genes detected in our samples, 644 were differentially expressed in the AF atrial samples with an adj.*p* < 0.05 and log_2_FC >1 or < -1 (Fig 1B). Of these, 265 genes were up-regulated and 379 were down-regulated. A complete list of DEGs is provided in D1 in S1 Dataset.

Pathway and gene ontology enrichment analysis was performed separately in up- and down-regulated genes (Fig 1C and 1D). We found that down-regulated genes were enriched for “integral component of plasma membrane”, “regulation of ion transmembrane transport” and “extracellular region”, while up-regulated genes were greatly involved in “extracellular space”, “nucleosome” and “mitotic nuclear division”. A full list of GO terms and KEGG pathways enriched in up- and down-regulated genes is available in D2 in S1 Dataset. We validated the RNA-seq results by testing the expression of eight genes in five AF and five control samples using qPCR quantification (S3 Fig in S1 File). The correlation between qPCR and RNA-seq results was high for all the genes tested.

We analysed the dataset to prioritize genes that may be involved in electrical remodelling, structural remodelling or genetic variants associated with AF in GWAS and familial studies. Comparative analysis of DEGs with AF-associated genes and genes involved in mechanisms of interest resulted in 57 up-regulated and 94 down-regulated genes, marked in D1 in S1 Dataset. Specifically, the analysis showed nine genes affected by AF-associated genetic variants, 14 down-regulated and three up-regulated ion channels/subunits, and nine upregulated genes associated with cardiac fibrosis and extracellular matrix components. Genes are listed in Table 2, including reference to published data reporting similar changes in AF patients.

**Table 2:** DEGs associated with AF and potentially involved in electrical and structural remodelling.

AF genes from GWAS and familial studies						
Symbol	Gene ID	log_2_FC	p-value	p-adjusted	Reference	Samples
*PHLDA1*	ENSG00000139289	2,94	5,79E-19	5,43E-16	[34]	-
*MYH7*	ENSG00000092054	1,97	6,01E-06	3,28E-04	[34]	-
*NAV2*	ENSG00000166833	1,18	2,68E-13	1,23E-10	[34]	-
*RPL3L*	ENSG00000140986	1,16	2,48E-15	1,70E-12	EV	-
*REC114*	ENSG00000183324	-3,01	5,71E-24	1,31E-20	[34]	-
*HCN4*	ENSG00000138622	-1,71	5,15E-11	1,50E-08	[34]	-
*KCNJ5*	ENSG00000120457	-1,10	1,85E-12	7,56E-10	[34]	-
*KCNN2*	ENSG00000080709	-1,06	1,86E-10	4,62E-08	[34]	-
*MYH6*	ENSG00000197616	-1,06	3,89E-05	0,0015	EV	-
**Ion Channel genes**						
*KCNQ4*	ENSG00000117013	1,09	0,0021	0,0343	-	-
*KCNQ3*	ENSG00000184156	1,36	1,58E-05	7,49E-04	-	-
*KCNA4*	ENSG00000182255	1,63	2,49E-09	4,50E-07	-	-
*KCNH7*	ENSG00000184611	-2,71	2,29E-07	2,16E-05	-	-
*KCNQ5*	ENSG00000185760	-1,79	1,24E-05	6,13E-04	-	-
*HCN4*	ENSG00000138622	-1,71	5,15E-11	1,50E-08	-	-
*CACNA2D2*	ENSG00000007402	-1,34	3,74E-11	1,13E-08	[36]	RA
*CACNA1G*	ENSG00000006283	-1,33	1,71E-07	1,67E-05	[36]	RA
*KCNK5*	ENSG00000164626	-1,32	2,45E-04	0,0066	-	-
*KCNK17*	ENSG00000124780	-1,30	5,34E-04	0,0120	-	-
*SCN2A*	ENSG00000136531	-1,29	8,71E-04	0,0174	-	-
*KCNB1*	ENSG00000158445	-1,20	3,96E-04	0,0096	-	-
*CACNA1D*	ENSG00000157388	-1,16	3,29E-05	0,0013	[36]	RA
*KCNJ5*	ENSG00000120457	-1,10	1,85E-12	7,56E-10	[36,37]	RA, LAA
*SCN1A*	ENSG00000144285	-1,10	0,0029	0,0429	-	-
*KCNN2*	ENSG00000080709	-1,06	1,86E-10	4,62E-08	[37]	LAA
*SCN3A*	ENSG00000153253	-1,05	0,0029	0,0423	-	-
**Extracellular Matrix/Cardiac Fibrosis**						
*NPPB*	ENSG00000120937	6,61	1,07E-49	8,98E-46	[36,38]	RA
*COLQ*	ENSG00000206561	2,90	2,30E-42	1,46E-38	[36–38]	RA, LAA
*NPPA*	ENSG00000175206	2,00	4,37E-24	1,10E-20	-	-
*TNC*	ENSG00000041982	1,87	0,0014	0,0245	[36,38]	RA
*COL21A1*	ENSG00000124749	1,66	9,52E-12	3,39E-09	-	-
*COL3A1*	ENSG00000168542	1,57	2,57E-06	1,62E-04	-	-
*COL12A1*	ENSG00000111799	1,53	3,01E-07	2,76E-05	[36]	RA
*COL1A1*	ENSG00000108821	1,52	1,11E-04	0,0036	[36,39]	RA, RAA
*ANGPTL2*	ENSG00000136859	1,09	5,47E-11	1,57E-08	[36,37,40]	RA, LAA

EV—Ensembl Variation 96; RAA–Right atrial appendage; LAA–left atrial appendage

### miRNAs are involved in remodelling of the right atrium

**Fig 2:**
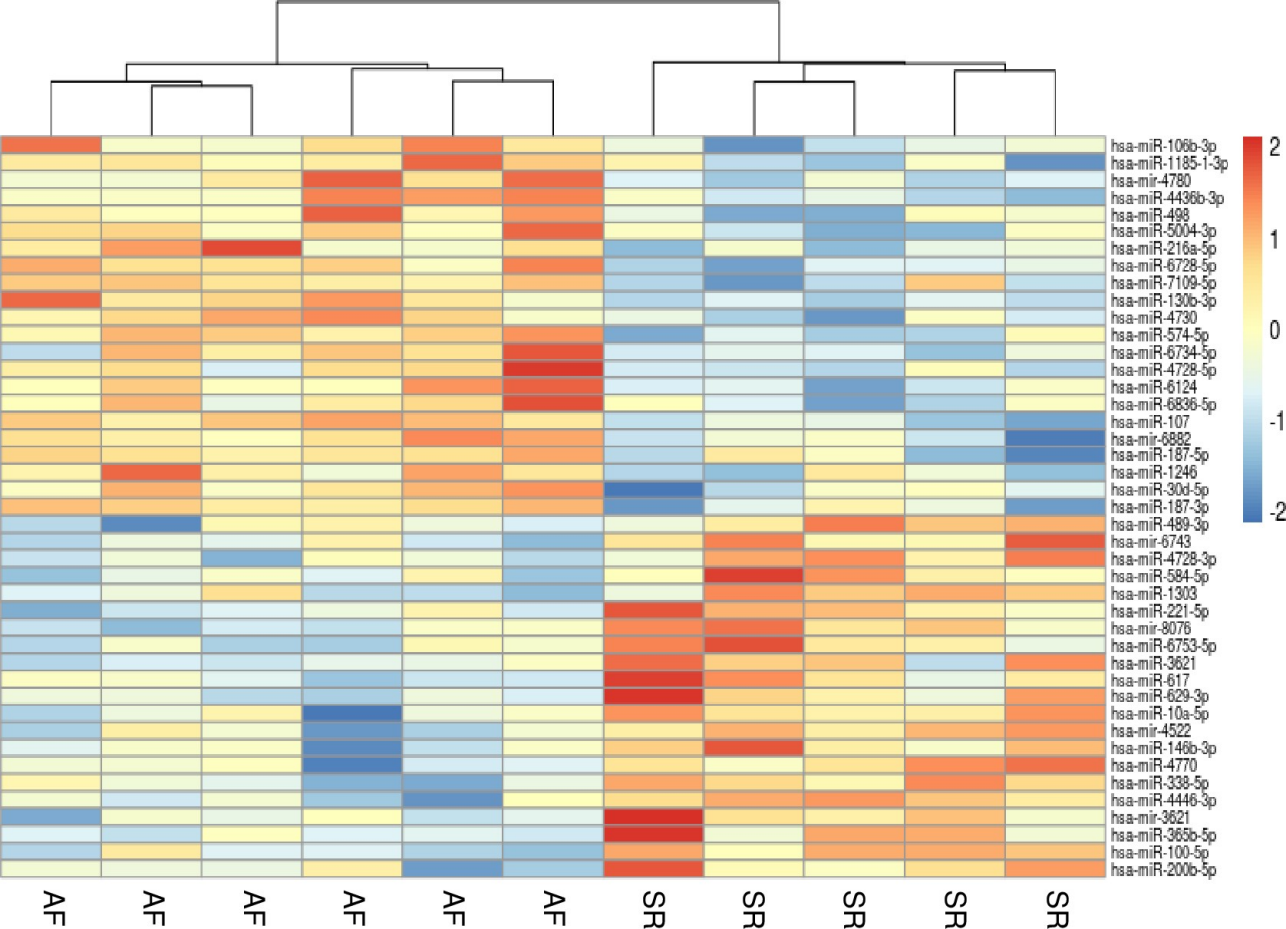
Micro RNAs expression analysis of right atrial biopsies from AF patients compared to controls (SR). Heatmap shows the 43 differentially expressed miRNAs in AF hearts. Columns represent samples and rows represent miRNAs. Red indicates increased expression, blue indicates decreased expression and yellow indicates low variation in relation to the mean expression.

Microarray experiments allowed us to investigate miRNAs expression profiles in RA biopsies. Different factors were expected to contribute to sample variability due to the heterogeneous profile of the study participants (AF duration, gender, age, alcohol consumption). We used PCA and sample-to-sample distance matrix to evaluate miRNAs expression variations between samples (S4 Fig in S1 File). We were able to identify a total of 43 miRNAs differentially expressed between AF and control subjects following a cut-off *p* < 0.01. Of those, 21 were down-regulated and 22 were up-regulated in AF patients, as shown in Fig 2. We validated the microarray experiments by qPCR quantification of eleven miRNAs in three AF and three control tissue samples (S5 Fig in S1 File). Altogether, qPCR results were in accordance with the microarray results. Average expression data from all subjects revealed that amongst the top 20 abundant miRNAs were miR-26a, miR-125b, let-7a/c and miR-23b-3p, thereby confirming the high expression levels in RA previously shown by others [41].

Amongst the differentially expressed miRNAs identified in this study, ten were reported previously as abnormally expressed in AF atrial tissues. miR-338-5, miR-10a-5p and miR-200b-5p were down-regulated in our AF patients with similar results reported in RA free wall and RA appendage (RAA) tissue samples of AF patients [30,31]. The same was observed with up-regulated miRNAs, namely miR-30d-5p, miR-216a-5p, miR-106b-3p, miR-130b-3p, miR-574-5p, miR187-5p and miR-187-3p, which were found to be up-regulated in the same human AF studies [30,31]. Increasing the *p*-value cut-off to 0.05, the number of miRNAs dysregulated in AF increased drastically to a total of 223 (D3 in S1 Dataset), including other commonly AF-associated miRNAs such as miR-15b, miR-26a-5p, miR-24-3p, miR-208a-5p and miR-193a-5p [15,30–32,42–44]. Nonetheless, some of the miRNAs most commonly reported dysregulated in AF, such as miR-1, miR-21 and miR-143, were unchanged in our study [45].

### Novel putative miRNA-gene interactions in AF

**Fig 3:**
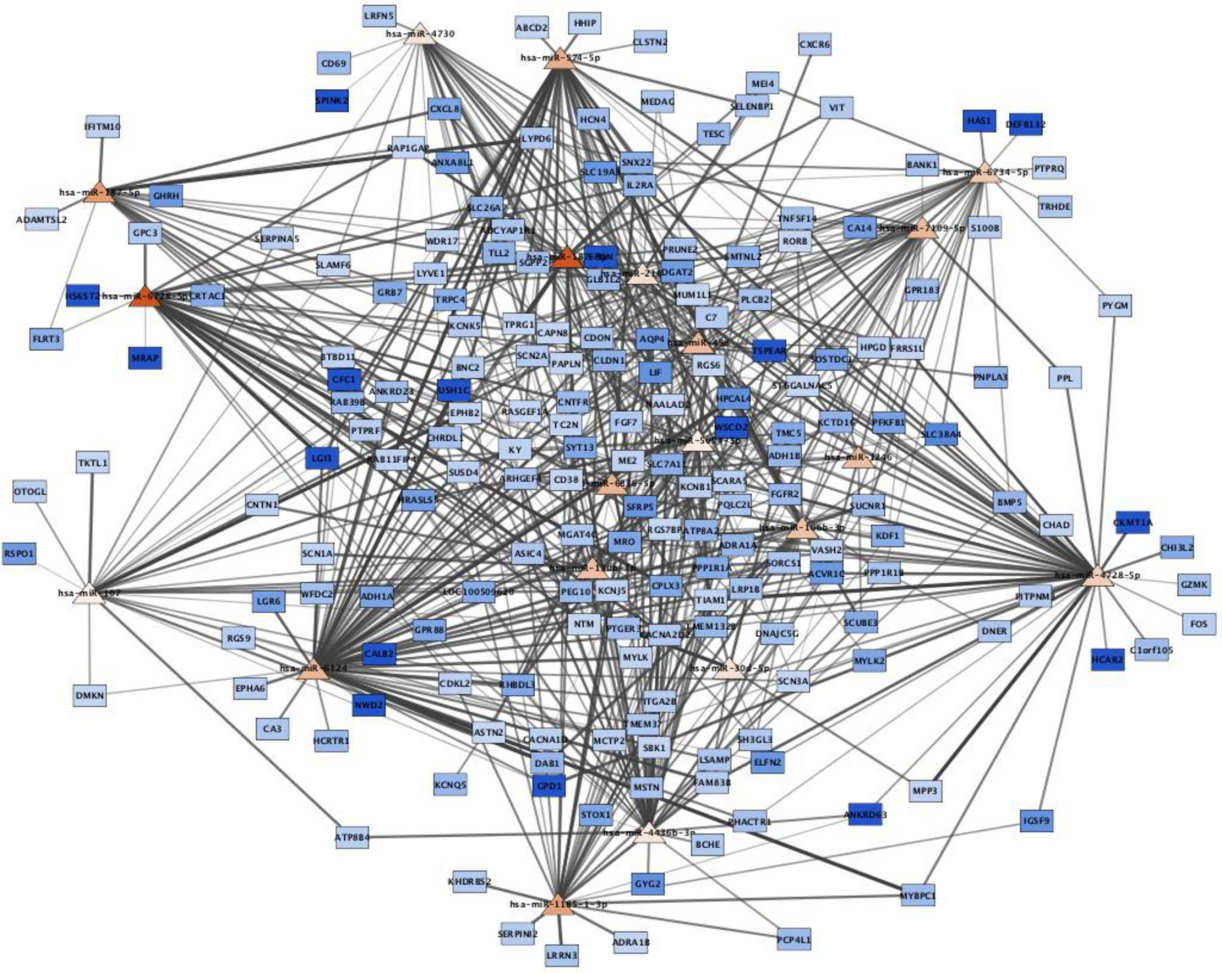
miRNA-gene regulatory networks build using Cytoscape. Network includes up-regulated miRNAs and candidate gene targets that were down-regulated in right atrial biopsies of AF patients compared to control subjects. The network helps to identify miRNAs targeting multiple DEGs and genes targeted by multiple miRNAs. Triangle nodes represent up-regulated miRNAs with red colour intensity according to FC. Rectangle nodes represent down-regulated genes with blue colouring according to FC. The darkness of the node to node edges correlates with the expression correlation value between a miRNA and its target gene. The darker the line, the closer to -1 is the correlation. All miRNA-gene pairs with correlation above -0.5 were excluded from the network. AF–atrial fibrillation; FC–fold-change.

The 43 dysregulated miRNAs were predicted to bind 166,790 3’-UTR sites of 15,213 unique target genes. Interaction with genes outside our DEGs list were excluded. miRNAs are expected to negatively regulate mRNA expression and therefore, expected to have opposite expression results. We analysed the expression correlation in all miRNA-mRNA interaction pairs using Spearman’s correlation coefficient. Negative correlation ranged -1 to -0.5 were selected. A total of 905 miRNA-mRNA pairs, including 37 miRNAs and 295 genes, passed the filtering (D4 in S1 Dataset). Fourteen of the miRNA-mRNA interactions were experimentally validated according to miRTarBase. We used Cytoscape to visualize the predicted regulatory networks [29]. Two networks were created, one with up-regulated miRNAs and corresponding down-regulated target genes (Fig 3) and a second network with down-regulated miRNAs and corresponding up-regulated target genes (S6 Fig in S1 File).

**Fig 4:**
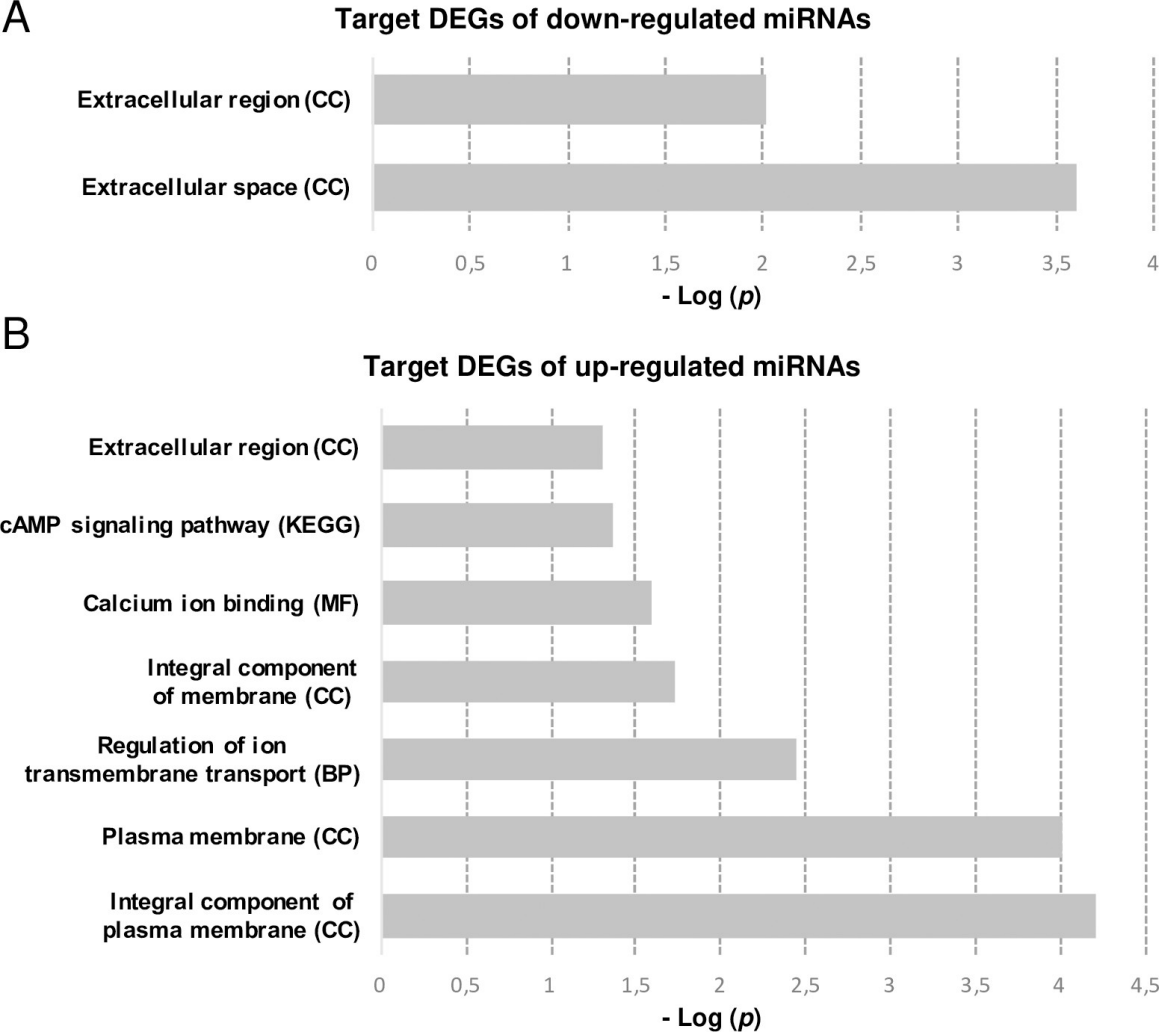
Gene enrichment analysis of micro RNA target genes using GO terms and KEGG. A. Analysis of up-regulated genes predicted to be targeted by down-regulated miRNAs. B. Analysis of down-regulated genes predicted to be targeted by up-regulated miRNAs. Enrichment cut-off was *p* <0.05 after Benjamini-Hochberg correction. BP–biological processes; CC–cellular component; GO–gene ontology; KEGG—Kyoto Encyclopedia of Genes and Genomes; MF–molecular function.

GO enrichment analysis revealed that our miRNA-gene interactions were enriched in eight categories (D5 in S1 Dataset). Down-regulated miRNAs were predicted to target genes enriched for “extracellular space” and “extracellular region” cellular components (Fig 4A). In up-regulated miRNAs, the most significant enrichments of target down-regulated genes were related to plasma membrane components, “regulation of ion transmembrane transport” and “calcium ion binding”. KEGG analysis showed enrichment in the “cAMP signalling pathway” (Fig 4B).

Amongst the up-regulated miRNAs targeting the most DEGs, we identified miR-4436b-3p, miR-4728-5p and miR-6124 as putative regulators of 57, 56 and 67 down-regulated genes. These miRNAs are poorly characterized. Enrichment analysis of the target genes of each miRNA, revealed that down-regulated genes targeted by miR-6124 are enriched for “calcium ion binding”. Considering the importance of calcium dysregulation in the triggering of AF and disease progression of AF [46], it renders miRNA-6124 a potential role in AF. Conversely, *AQP4* and *RGS6* were the down-regulated genes predicted to be regulated by the higher number of overexpressed miRNAs, ten and thirteen respectively.

**Fig 5:**
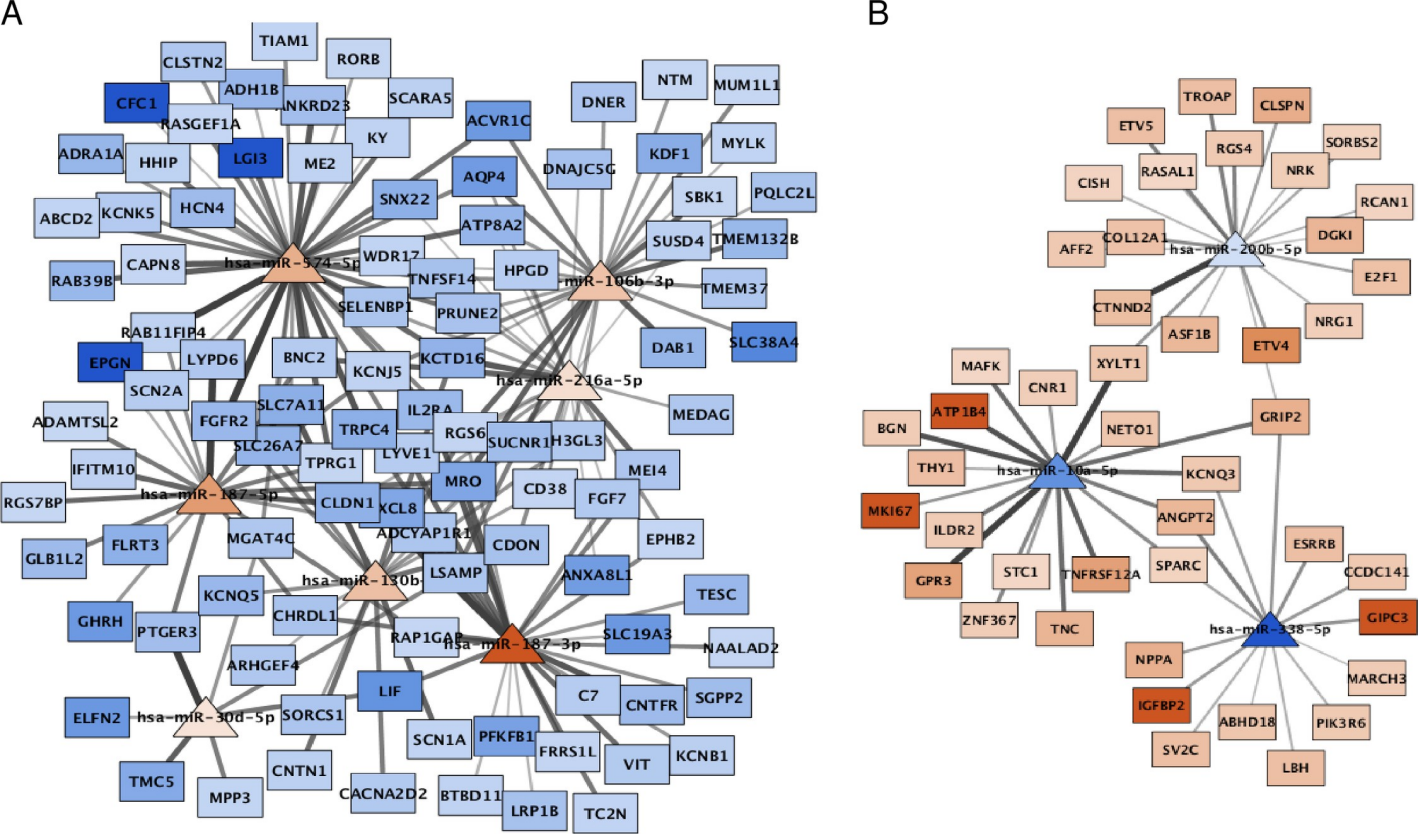
AF related miRNA-gene regulatory networks build using Cytoscape. MiRNAs previously associated with AF were selected to create a subset of the regulatory networks. A. Up-regulated miRNAs and candidate gene targets down-regulated in right atrial biopsies of AF patients compared to control subjects. B. Down-regulated miRNAs and candidate gene targets up-regulated in AF patients. Triangle nodes represent miRNAs and rectangle nodes represent target genes. Red colour intensity varies according to expression increase in FC and blue colouring according to decrease in FC. Edges connecting miRNA and genes are coloured according to Spearman correlation of expression data. The darker the line, the closer to -1 is the correlation. AF–atrial fibrillation; FC–fold-change.

Lastly, we selected a subset of interactions including exclusively miRNAs that were previously shown to be dysregulated in AF tissues. The AF related regulatory networks are shown in Fig 5.

### AF genomic elements co-localize in high-density regions

**Fig 6:**
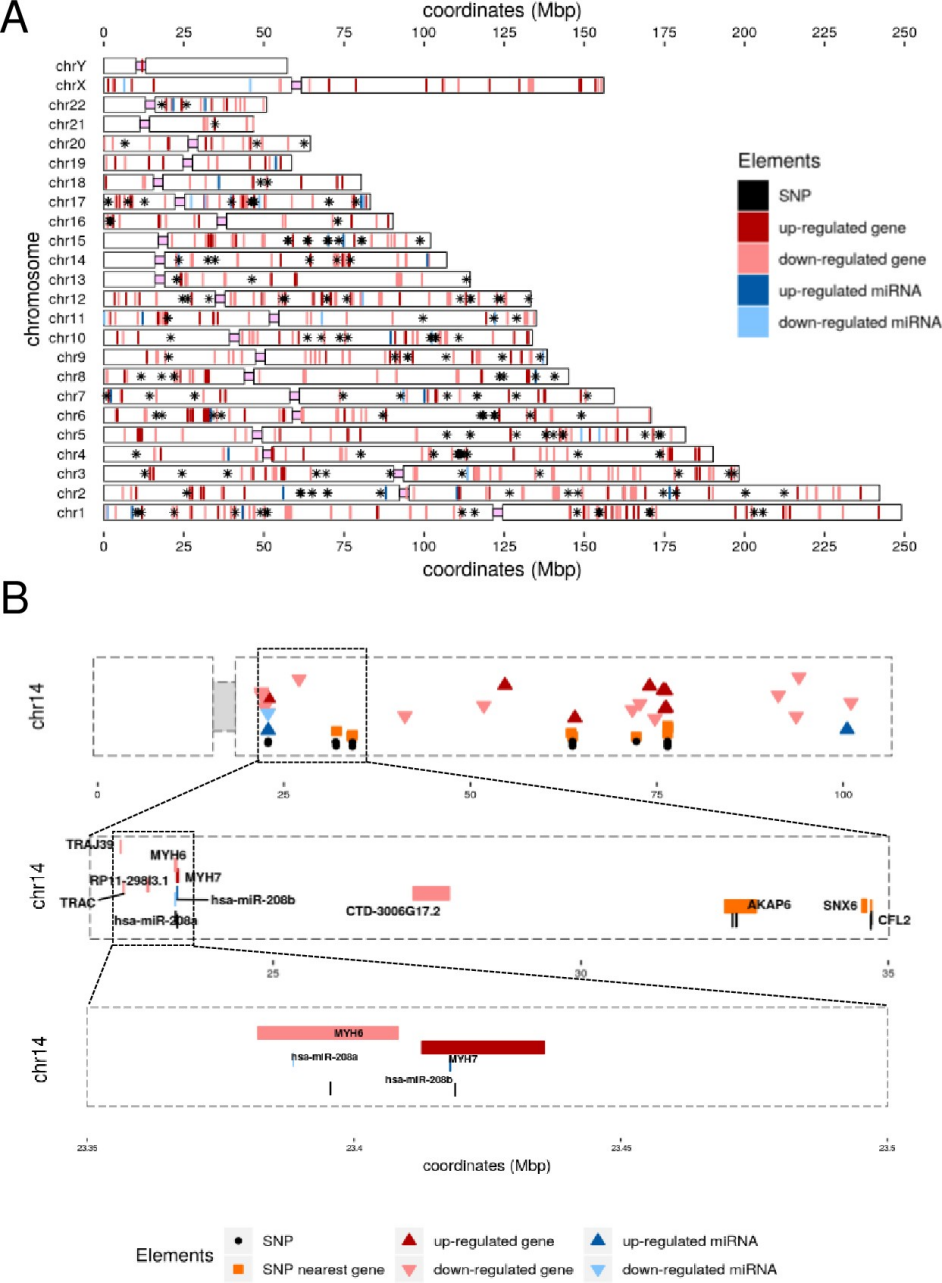
Chromosome enrichment of AF related genetic elements. Enrichment includes AF related SNPs and differentially expresses genes and miRNAs in right atrium biopsies of AF patients compared to control subjects. A. Chromosome plots showing the genomic location of all 1008 elements included in the study. B. High-density cluster identified in the q arm of chromosome 14 including genes *MYH6* and *MYH7*, miR-208a/b and two AF-associated SNPs. AF–atrial fibrillation; SNPs–single nucleotide polymorphisms; Mbp–million base pairs.

Genetics factors contributing to AF involve mono- and polygenic mutations and/or dysregulation of genes and non-coding RNAs, resulting in disrupted protein expression. Additionally, expression regulators, such as methylation and chromatin modifications, can regulate expression of entire sets of genes located in proximal genomic regions. To assess a possible chromosomal clustering of our dysregulated miRNAs and DEGs with previously reported AF-associated genetic variants, we analysed their relative genomic localisation. A total of 1008 genetic components were integrated in the analysis, including 43 DE miRNAs, 644 DE genes and 321 SNPs. Fig 6A illustrates a chromosome plot with the location of all genetic components included in our study. Chromosomes 1 and 6 show the highest density of genetic elements after normalization, with 107 and 78 elements respectively. Zooming into clusters with five or more genetic elements, we have identified seven high-density clusters located in chromosomes 2, 6, 11, 14, 15, 16 and 17. For a full list of clusters, genetics elements and genomic locations, see D6 in S1 Dataset.

An example locus is shown in Fig 6B. The figure represents cluster 580 located in chromosome 14 which includes rs422068, rs28631169, *MYH6*, *MYH7*, miR-208b and miR-208a. Another cluster, number 298, is located in the p arm of chromosome 6 and includes the highest number of novel genes associated with AF (S7 Fig in S1 File). This cluster is part of the major histocompatibility complex (MHC) class III gene cluster and contains five up-regulated genes from our RNA-seq experiments. The MHC III cluster is heterogeneous and poorly characterized. The MHC III region is composed by more than 60 genes encoding signalling molecules involved in inflammatory response and a variety of different cell communication processes [47].

Furthermore, we analysed the genomic location of dysregulated miRNAs and genes to assess possible co-expression patterns. 22 of our 42 differentially expressed miRNAs were located in either intronic or exonic regions. The expression of both miRNA and host genes was changed in the same direction in ten of the 22 miRNAs. In four of those cases, changes were significant for both the miRNA and the gene. These findings support previous studies showing that transcription of intronic and exonic miRNAs is regulated by the same promoter as their host genes [48,49].

## Discussion

Transcriptome analysis is a powerful tool to help identify new molecules and pathways of disease as it allows the simultaneous analysis of coding and non-coding RNAs. To date, few studies have applied RNA-sequencing to study transcriptome changes in tissue samples from AF patients [50,51]. In this study, we analyse the expression of genes and miRNAs in RA biopsies to investigate differences between in MVR patients with and without AF. The strength of our study is that we assessed miRNA and gene expression from the exact same tissue samples and pool of extracted RNA, thereby increasing the likelihood of selecting relevant miRNA-mRNA pairs.

We detected changes of expression in 14 cardiac ion channels that can generate electrical conductance disturbances. Notably, genes *KCNJ5*, *KCNN2* and *HCN4*, encoding the inward rectifier K^+^-channel subunit K_ir_3.4 (GIRK4), the small conductance calcium activated potassium channel member 2 K_Ca_2.2 (SK2*)* and the hyperpolarization activated cyclic nucleotide gated potassium channel 4 (*HCN4*), respectively, were down-regulated in AF. These findings are in line with other transcriptional studies in different atrial samples [36,37]. The three channels have also been reported in AF GWAS studies. Down-regulation of *KCNJ5* is believed to represent a compensatory mechanism to counteract shortening of the atrial effective refractory period seen in persistent AF [37]. In the heart, *KCNN2* is predominantly expressed in the atria. Our findings of reduced *KCNN2* expression in AF are consistent with Skibsbye et al. who reported *KCNN2* down-regulation and reduced functional importance of the channel in chronic AF [52].

Genes associated with increased cardiac fibrosis and extracellular matrix were upregulated in AF patients. This is an indicator of structural remodelling in hearts of AF patients mediated by interstitial fibrosis due to accumulation of collagen fibres and fibroblasts in the extracellular matrix. The fibrotic processes can be induced via activation of the TGF-ß1/Smad3 signalling pathway with increase *COL1A1* and *COL3A1* expression [53]. Unfortunately, we were unable to investigate interstitial fibrosis in our AF patients, due to limited tissue availability. Lastly, we found *NPPB* to be up-regulated by almost 7-fold in our AF patients. *NPPB* encodes the natriuretic peptide B protein secreted by cardiac myocytes. We hypothesize that its up-regulation might be a response to cardiac fibrosis due to its anti-fibrotic function [54]. Overall, the changes in genes expression described here are in line with the electrical, contractile, and structural remodelling characteristic of AF hearts. We hypothesize that the more severe remodelling of the heart in certain MVR patients can be the key factor to create the trigger and subtract for development and maintenance of AF. The contribution of structural remodelling to AF susceptibility was also described by in two previous studies [55,56], both showing that more severe fibrosis in RA and LA was associated with increased susceptibility to develop AF in patients with concomitant heart disease and after cardiac surgery.

As negative regulator of gene expression, miRNAs bind to the 3’-UTR of target genes to inhibit protein production, by preventing translation by ribosomes and promoting mRNA degradation [11]. Importantly, a gene simultaneously targeted by multiple miRNAs is more likely to be regulated by miRNAs. Likewise, overexpressed miRNAs that target multiple genes are more likely to have an important role in a specific tissue [57]. Hence, it is important to study the collective action of miRNAs in disease. In the present study, differentially expressed miRNAs include ten miRNAs previously reported as changed in AF tissues, but with little known about their functional role. We built an AF related regulatory network including 905 miRNA-target gene pairs that can play a role in the development of AF in MVR patients.

Aquaporin 4 (*AQP4*) and regulator of G-protein signalling 6 (*RGS6*) were the down-regulated genes predicted to be targeted by the highest number of DE miRNAs. *AQP4* contributes to maintenance of water and electrolyte balance in aging hearts and its decreased expression might compromise homeostasis in cardiac cells [58]. *RGS6* is involved in modulation of parasympathetic regulation of heart rate. Down-regulation of *RGS6* can contribute to electrical remodelling and shortening of the action potential by increasing the activation time of the G protein-coupled inwardly rectifying K^+^ K_ir_3.1/3.4 (GIRK) channel, resulting in a large K^+^ current and membrane hyperpolarization [59,60].

MiR-338-5p targeted 14 of our DEGs, including *IGFBP2*, *LBH* and *NPPA*. *IGFBP2* and *LBH* have both been reported as up-regulated in three different AF studies and they are increased by 8 and 3 FC in our RA samples, respectively [36–38,40]. Mutations in *NPPA* have been associated with familial AF [61]. *NPPA* encodes the natriuretic peptide A protein, a circulatory hormone released from atrial cardiomyocytes upon stretching. The NPPA protein plays a role in cardiac electrophysiological function, where it shortens the atrial effective refractory period and conduction velocity in human hearts by inhibiting sympathetic and parasympathetic activity or through direct regulation of cardiac ion channels [62–64]. MiR-338-5p was also reported as down-regulated in RAA of AF patients with mitral stenosis [30]. This miRNA has also been implicated in tumor progression and reported to alleviate lung fibrosis [65,66].

MiR-130b-3p was upregulated in RA samples in line with a previous study [31]. MiR-130b-3p is predicted to bind 15 of our down-regulated genes, amongst those there are *CACNA2D2* and *SLC7A11* which have been reported as down-regulated in AF transcriptomics studies [36,38,67]. *CACNA2D2* is a subunit of the voltage-gated calcium channel that modulates the calcium signalling response in cardiomyocytes and induces arrhythmia when down-regulated [68].

Genomic co-localization analysis helped mark regions of interest in the long list of DE genes and miRNAs. A high-density cluster in chromosome 14q11.2 marked a genetic locus that includes genes *MYH6*, *MYH7*, miR-208a, miR-208b and SNPs rs422068 and rs28631169 within 20 kbp of distance. We find miR-208a down-regulated in AF patients by increasing the array analysis *p* threshold to 0.05. The same was reported in RAA from AF patients and atria of ventricular tachypaced dogs [30,32,69]. MiR-208a-3p was described by Li et al. to regulate the expression of connexin 40 in the heart, resulting in electrical impulse propagation defects between adjacent cardiac myocytes [70]. Furthermore, miR-208a-3p is believed to generate protective effects in myocardial infarction and it could become a potential treatment for structural remodelling in AF [71]. Genetic deletion of miR-208a in mouse hearts interferes with cardiac conduction, with lack of P waves in ECG recordings and an AF like phenotype [72]. MiR-208a is encoded by an intron of the *MYH6*, which is also down-regulated in our samples, therefore suggesting that miR-208a is co-expressed with its host gene. *MYH6* is an important protein in cardiac muscle fibre composition, involved in muscle contraction mechanisms. Both miR-208a and *MYH6* are expressed predominantly in the atrial chambers. *MYH7* and miR-208b are up-regulated in our RNA-seq studies by 4 and 3.5 FC, suggesting once again co-expression. *MYH7* is mainly expressed in the ventricular chambers and is part of sarcomere formation, therefore involved in cardiac contraction mechanisms. Several *MYH7* mutations have been reported in cardiac muscle disorders, including AF [73]. MiR-208b was reported up-regulated in isolated myocytes from chronic AF patients and is involved in calcium homeostasis and contractile remodelling [74]. In conclusion, our study emphasizes the significance of chr14q11.2 as a AF susceptibility locus.

### Study limitations

The study cohort size is modest and challenges the assessment of the effect of confounding factors in the results. Furthermore, it is important to mention that different studies investigating miRNA and gene expression in atrial samples from AF patients overlap poorly [45]. These inconsistencies might reflect 1) heterogeneity between tissue samples (location and collection method), 2) AF type and duration, 3) variation in patient cohorts (comorbidities, life-style, age, medication), and 4) the experimental technique selected to study transcript changes. In fact, our RNA-seq results overlap best with gene expression profiling studies from Jiang et al. using similar tissue samples and experiments [38]. A continuous effort from experts to study larger cohorts and collect similar datasets will allow a combined analysis of multiple samples from AF patients and help on accurate selection of target genes and regulatory networks involving miRNAs.

The *in silico* miRNA-target gene predictions provide qualified results which are generally accepted by research. However, these interactions require further validation and functional studies to clarify the pathophysiological role of such interactions, such as luciferase assays and *in vitro* miRNA transfections.

Lastly, the functional role of each DEG in AF is difficult to estimate since many genes are not yet characterized and we miss information on cell type specificity. For instance, we see down-regulation of *KCNQ5* in our AF samples, a gene known for being mainly expressed in neuronal, skeletal and smooth muscle cells and therefore, not expected to interfere with cardiac electrical conductance [75]. Single-cell gene expression studies would allow us to detect the cell population creating the change, giving a better idea of the mechanism in which each DEG is involved.

## Conclusions

By combining miRNA and gene expression data from RA of MVR patients with AF, we were able to create a novel miRNA-mRNA regulatory network, providing new insights into the mechanism predisposing certain MVR patients to develop and maintain AF. Down-regulation of ion channel genes and up-regulation of extracellular matrix genes summarize the major changes creating a subtract for AF. Lastly, we identified a high-density AF loci in chromosome 14q11.2.

## Supporting information

S1 File Supplementary methods and S1–S7 Figs. (PDF)

S1 Dataset Excel file with S1–S6 Data. (XLSX)
