# Supplementary material for: Investigating gene-microRNA networks in atrial fibrillation patients with mitral valve regurgitation
Source: PLoS One. 2020 May 11;15(5):e0232719. doi: 10.1371/journal.pone.0232719 (PMC7213724; doi:10.1371/journal.pone.0232719)
Supplement: S1 File — (PDF) [file pone.0232719.s001.pdf]

Investigating gene-microRNA networks in atrial fibrillation patients with mitral valve regurgitation

Joana Larupa Santos[1], Ismael Rodríguez[1], Morten S. Olesen[1,2], Bo Hjorth Bentzen[1*¶], Nicole Schmitt[1*¶]

[1] Department of Biomedical Sciences, Faculty of Health and Medical Sciences, University of Copenhagen, Copenhagen N, Denmark;

[2] Laboratory for Molecular Cardiology, Department of Cardiology, The Heart Centre, Rigshospitalet, University Hospital of Copenhagen, Copenhagen Ø, Denmark.

[*] Corresponding authors

E-mail: bobe@sund.ku.dk (BHB) and nschmitt@sund.ku.dk (NS)

[¶]These authors contributed equally to this work.

Supplementary methods

RNA preparation

Total RNA including small RNAs was extracted from RA biopsies of six AF patients and six control patients. Briefly, tissue samples were homogenized in QIAzol reagent (QIAGEN, Maryland, USA) using a Precellys 24 homogenizer (Bertin Technologies, Montigny-le-Bretonneux, France). Samples were DNase treated and RNAs were isolated using the miRNeasy kit (QIAGEN, Hilden, Germany) according to manufacturer's instructions. RNA concentration was measured in a NanoDrop 2000 (ThermoScientific, Wilmington, USA) and quality was assessed using the RNA 6000 Nano RNA assay in a 2100 Bioanalyzer (Agilent Technologies, Santa Clara, CA, USA). RNA samples with a RIN > 6 were used in further experiments.

RNA-sequencing

We used a total of 50 ng of extracted RNA per sample to perform RNA-sequencing experiments. First, ribosomal RNAs were depleted from total RNA using the Ribo-Zero rRNA removal kit (Illumina, San Diego, California, USA). Barcoded cDNA libraries were prepared using the TruSeq Stranded Total RNA Library Prep Kit (Illumina, San Diego, California, USA) following manufacturer's protocol. Libraries were then sequenced on an Illumina HiSeq 2500 with four library samples per lane (125 bp pair-end reads). The control sample HS24 was not used due to low amount of input RNA. The average number of reads per sample ranged from 71 to 82 million. This work was performed at the Next Generation Sequencing Service Facility, Center for Genomic Medicine, Righospitalet, Denmark (www.genomic-medicine.dk).

Transcriptome sequencing analysis

The raw paired-end reads were aligned to the human GRCh38 reference transcriptome using the Kallisto pseudoaligner with default options [1]. The Kallisto index was built using the protein-coding cDNA reference combined with the non-protein coding transcripts (ncRNA) reference. Both references were obtained from Ensembl (ftp://ftp.ensembl.org/pub/release-95/fasta/homo_sapiens/). Kallisto quantifies the

abundance of reads on transcript level. On average, 54.24% of the reads mapped to the transcriptome yielding an average of 41,868,553 mapped reads per sample. The resulting quantification files were imported to R using tximport (Bioconductor) and transformed to transcript per million (TPM) for downstream analysis. Transcript counts were collapsed to gene level using BioMart (Bioconductor). Genes with TPM < 1 between all samples were discarded, leaving a final gene set of 44,852 ensemble gene IDs. Differentially expressed genes in AF compared to control tissue samples were obtained using the Bioconductor package DEseq2 [2]. DEseq2 uses the Wald test for significant testing and the Benjamini-Hochberg method to control for FDR. Genes with false discovery rate (adj.*p*) < 0.05 and log2fold-change (FC) above 1 or below -1 were considered differentially expressed genes (DEGs). Principal component analysis (PCA) and unsupervised hierarchical clustering of sample-to-sample distance matrixes were used to analyse sample clustering according to transcriptomic similarities. Heatmaps and volcano plots were used to visualize gene expression differences.

Validation of RNA-sequencing by quantitative PCR

Qualitative polymerase chain reaction (qPCR) was performed to confirm the reliability of the RNA-sequencing data. Eight genes were tested using the exact same isolated RNA samples from the RA of five AF patients and five control subjects. Reverse transcription was performed from 1000ng of total RNA using the Precision nanoScript2 Reverse Transcription kit (PrimerDesign, Southampton, United Kingdom) according to manufacturer's instructions. The expression of the genes *KCNA4, ACTN2, KCNK3, NANOG, TNNT2, KCNQ5, KCNJ5* and *KCNB1,* was measured using Taqman double dye probes and PrecisionPLUS MasterMix with ROX (PrimerDesign, Southampton, United Kingdom) in a light cycler CFX Connect Real-Time System (BIO-RAD, Hertfordshire, UK). Samples were tested in triplicates. The following qPCR method was used: 95°C for 2 min followed by 40 cycles of 95°C for 15 s and 60°C for 1 min. The Taqman double dye probes for genes *YWHAZ* (PrimerDesign, UK) and *RPL13A* (PrimerDesign, UK) were used as reference to normalize the results. Threshold cycle (C*t*) values were obtained from Bio-Rad CFX96 Manager 3.0 software. Relative expression and fold-change values were calculated using the $2^{-\Delta\Delta Ct}$ method comparing AF to control samples.

Primer sequences:

Gene	Forward primer	Reverse Primer	Prod. length	Accession number
KCNA4	5'-AGGAGGTGAAGTTCTATCAGTTGG-3'	5'-CCTGTCTTCCTCTTCTCTCACAAA-3'	86	NM_002233.3
ACTN2	5'-GTTCATTGTCCACAGCATTGAG-3'	5'-CACCTTCTCCACCTCGTTCT-3'	130	NM_001103
KCNK3	5'-CCCCACTCCCCTTCCTCAT-3'	5'-CTCCTTCTTTCTGTCCTGCTTTC-3'	139	NM_002246
NANOG	5'-GCTGTGTGTACTCAATGATAGATTT-3'	5'-GAGGTTCAGGATGTTGGAGAG-3'	85	NM_024865
TNNT2	5'-CAAAGCCCAGGTCGTTCAT-3'	5'-GCAACTCATTCAGGTCCTTCT-3'	114	NM_000364
KCNQ5	5'-AGCAGAACATGAGACCACAGA-3'	5'-CCGAAGGACCTGTTGATAGATG-3'	124	NM_019842
KCNJ5	5'-GGGCTGACACCTAGAGAGAA-3'	5'-CATCCCAGACTAGAGGTTTGAG-3'	116	NM_000890
KCNB1	5'-AGATCCTTGCCATAATTTCCATCAT-3'	5'-GCTGGGGGTTGTCTGTGG-3'	120	NM_004975

Reference Gene	Species	Accession number	Anchor Nucleotide	Product length
YWHAZ	Homo Sapiens	NM_003406	2585	150bp
RPL13A	Homo Sapiens	NM_012423	727	223bp

MiRNAs microarray

Microarrays were performed using the exact same RNA samples isolated for the RNA-seq experiments A total of 130 ng RNA per sample was used for each array. After labelling with FlashTag Biotin HSR RNA Labeling Kit (Affymetrix, Santa Clara, USA), mature miRNA transcripts were hybridized to the GeneChip® miRNA 4.0 Array (Affymetrix, Santa Clara, USA) and scanned. This work was performed at the Microarray Service Facility, Center for Genomic Medicine, Righospitalet, Denmark (www.genomic-medicine.dk).

The raw data from the CEL files generated by the Affymetrix's miRNA array was normalized using the robust multi-array average (RMA) method [3]. Microarray quality metrics showed one control sample (HS24) as an outlier (Supplementary Figure S1). The sample was removed from the analysis. The array data was then filtered to include only human probes in the analysis output with a final number of 4,202 mature

human miRNAs. The FC values and p-values (*p*) of expression changes were calculated using the Limma package in R/Bioconductor project [4]. FC values between the two groups were log2 transformed. A cut-off *p* lower than 0.01 was used for selection of differentially expressed miRNAs. No FC cut-off was applied. Samples and differentially expressed miRNAs were subjected to unsupervised hierarchical clustering and plotted as a heat map using the pheatmap package from CRAN.

Validation of microarray by qPCR

qPCR experiments were performed to confirm the reliability of microarray data, including eleven mature miRNAs that were both up- and down-regulated in the microarray assay. Three RNA samples from patients with AF and three from control subjects were tested in triplicates. cDNA was synthesized from 10 ng total RNA using the Universal cDNA Synthesis kit II (Exiqon, Wobrun, Massachusetts, USA). The qPCR reactions were performed using commercial miRCURY LNA™ Universal RT microRNA PCR primer and the ExiLENT SYBR® Green master mix (Exiqon, Wobrun, Massachusetts, USA) according to manufacturer's instructions. Experiments were performed in a light cycler CFX Connect Real-Time System (BIO-RAD, Hertfordshire, UK) using the following method: denaturation step at 95°C for 10 min, followed by 40 amplification cycles at 95°C for 10 s and 60°C for 1 min with a ramp-rate of 1.6°C/s. Fluorescence was measured at the end of each cycle.

The following miRNAs were used for validation: miR-143-5p, miR-192-5p, miR-187-3p, miR-208b-3p, miR-338-5p, miR-335-5p, miR-432-5p, miR-490-5p, miR-499a-5p, miR-503-5p and miR-92b-3p. miRNA-16-5p and miR-103a-3p were used as references to normalize the results. Threshold cycle (C*t*) values were obtained from Bio-Rad CFX96 Manager 3.0 software. Relative expression and FC were calculated using the $2^{-\Delta\Delta Ct}$ method to compare the expression of miRNAs in AF samples and control samples.

References

1. Bray NL, Pimentel H, Melsted P, Pachter L. Near-optimal probabilistic RNA-seq quantification. Nat Biotechnol. 2016;34(5):525–7.

2. Love MI, Huber W, Anders S. Moderated estimation of fold change and dispersion for RNA-seq data with DESeq2. Genome Biol. 2014 Dec 5;15(12):550.

3. Irizarry RA, Hobbs B, Collin F, Beazer-Barclay YD, Antonellis KJ, Scherf U, et al. Exploration, normalization, and summaries of high density oligonucleotide array probe level data. Biostat Oxf Engl. 2003 Apr;4(2):249–64.

4. Phipson B, Lee S, Majewski IJ, Alexander WS, Smyth GK. Robust hyperparameter estimation protects against hypervariable genes and improves power to detect diferential expression. Ann Appl Stat. 2016 Jun;10(2):946–63.

Supplementary figures

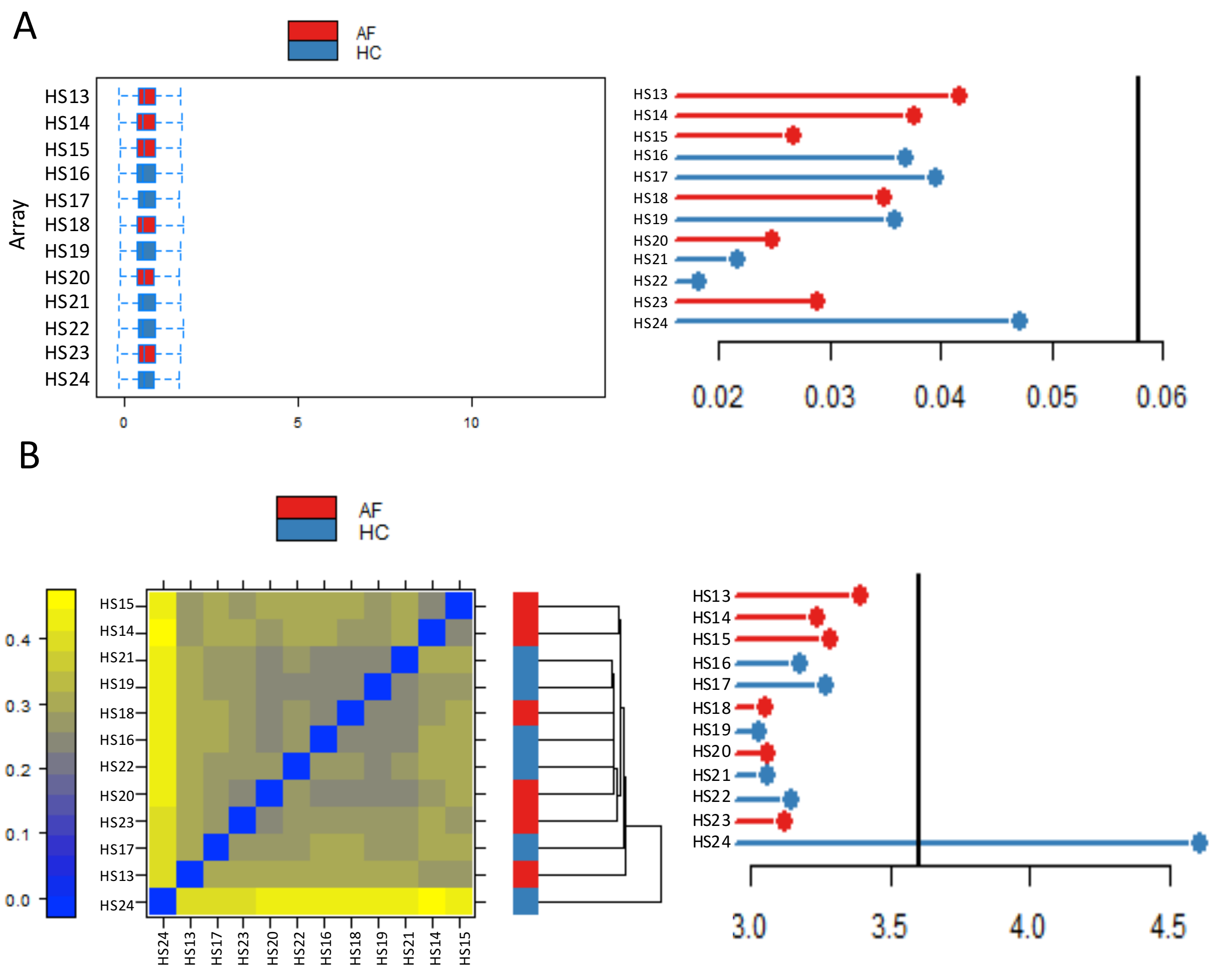

S1Fig. – Microarray quality metrics. A. Boxplots representing normalized array signal intensity distributions of each sample and outlier detection performed by computing the Kolmogorov-Smirnov statistic K_a between each array distribution. None of the arrays exceeded the outlier threshold indicated by the vertical line. **B**. False color heatmap of the distances between arrays. The distance between two arrays is computed as the mean absolute difference between the data of the arrays. Pattern in the plot can indicate clustering of the sample array due to biological similarities or unwanted experimental variation. Outlier detection is determined by looking the sum of the distances of one array to all other arrays. Basds on the distribution of the values across the arrays, sample 12 (HS24) exceeded the threshold and was considered an outlier.

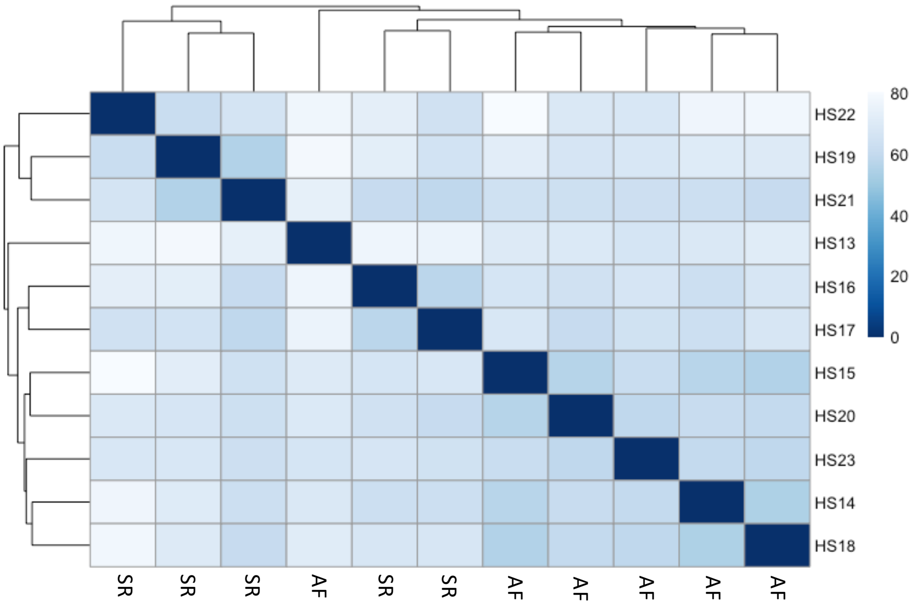

S2 Fig. – RNA-sequencing studies of right atrial tissue samples from AF patients and control subjects (SR). Heatmap of sample-to-sample distance of count matrixes showing similarities between samples. Dark blue represents a low distance and therefore high similarity. AF – atrial fibrillation

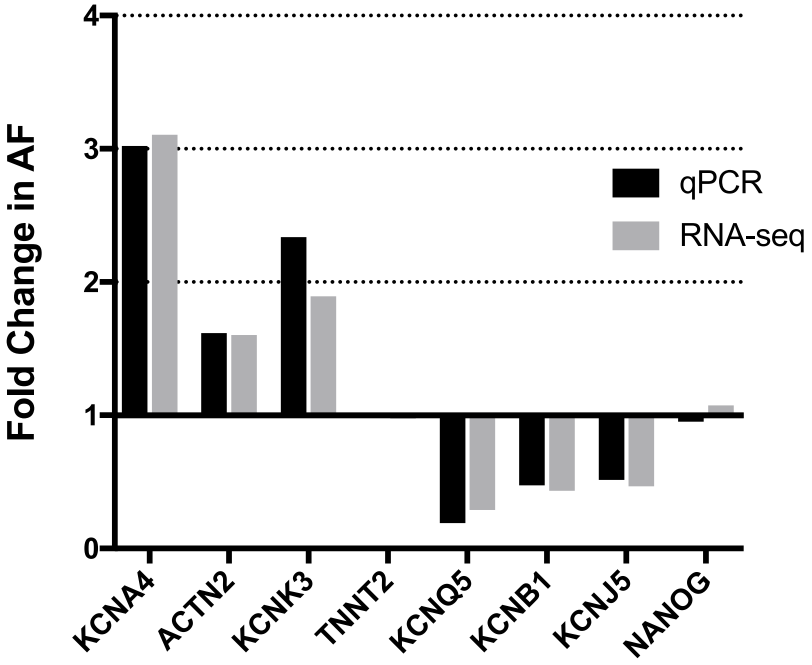

S3 Fig. – Quantitative real-time polymerase chain reaction (qPCR) of eight genes in AF patients compared to SR controls (n=5) used to validate RNA-sequencing results. The bar plot shows good correlation between both techniques, allowing the validation of RNA-sequencing data. qPCR results were normalized to the expression of *YWHAZ* and *RPL13A*. AF – Atrial Fibrillation; SR – sinus rhythm

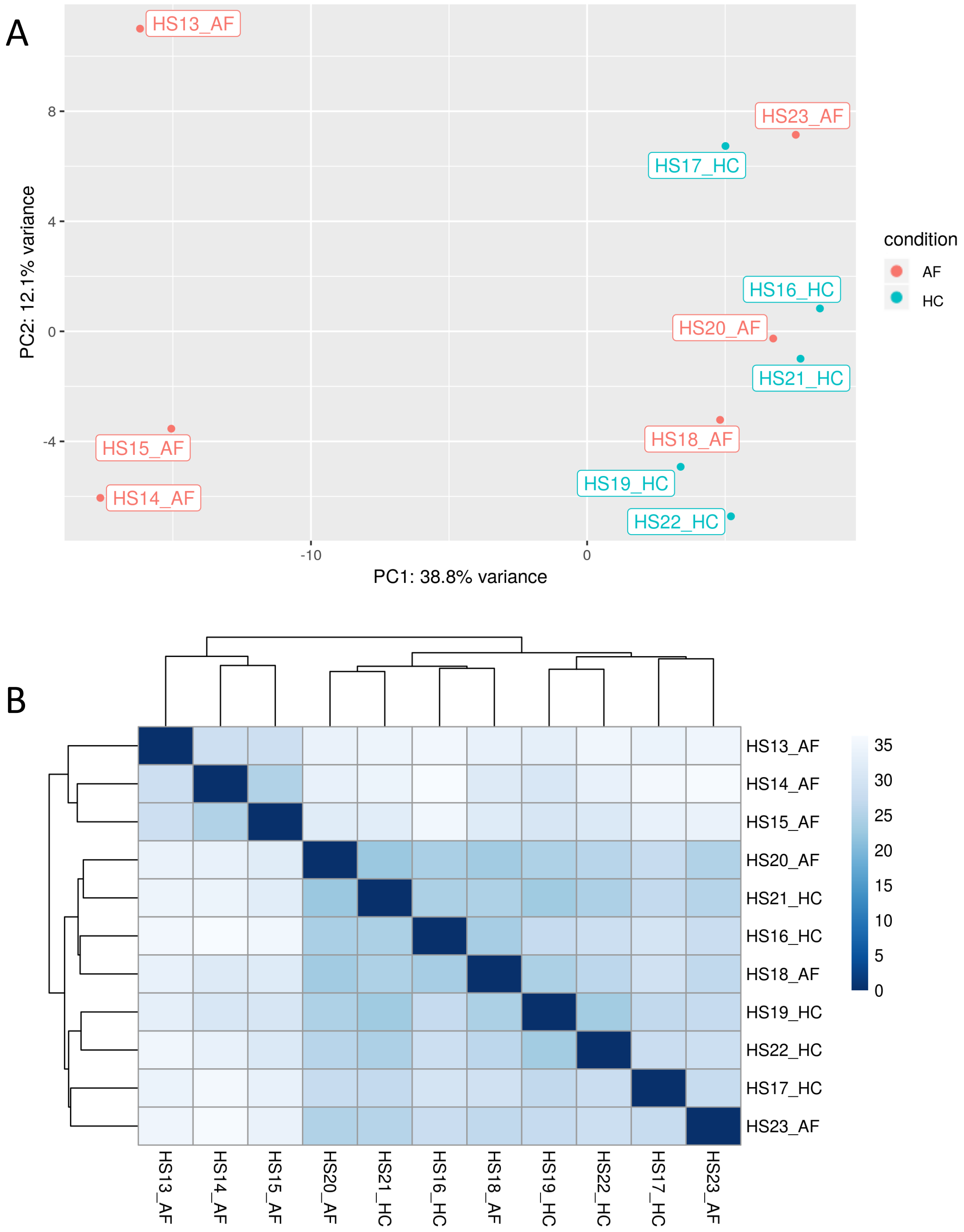

S4 Fig. – microRNA microarray of right atrial tissue samples from AF patients and controls (HC). A. Principal component analysis (PCA) showing the overall effect of variances between samples. None of the known patient characteristics in Table 1 contribute to the variation observed in the AF samples (13, 14, 15 and 18, 20, 23). **B**. Heatmap of sample-to-sample distance matrix showing similarities between sample. Dark blue represents a low distance and therefore high similarity. AF – atrial fibrillation; SR – sinus rhythm

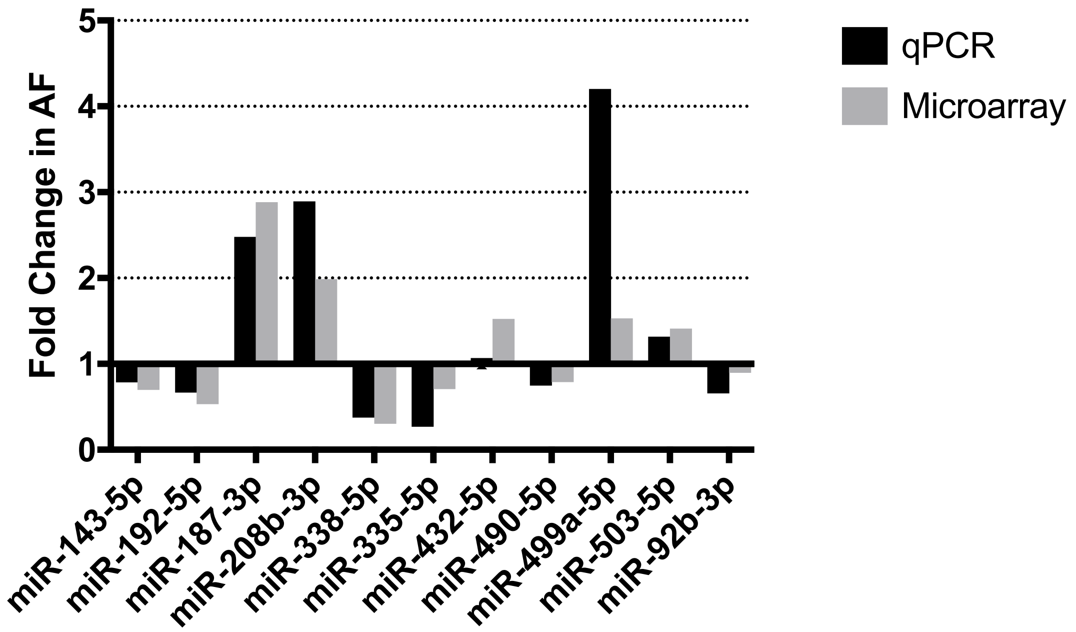

S5 Fig. – Quantitative real-time polymerase chain reaction (qPCR) of 11 miRNAs in AF patients compared to SR controls (n=3) used to validate microarray results. The bar plot shows good correlation between both techniques, allowing the validation of microarray data. qPCR results were normalized to the expression of miRNA-16-5p and miR-103a-3p. AF – Atrial Fibrillation; SR – sinus rhythm

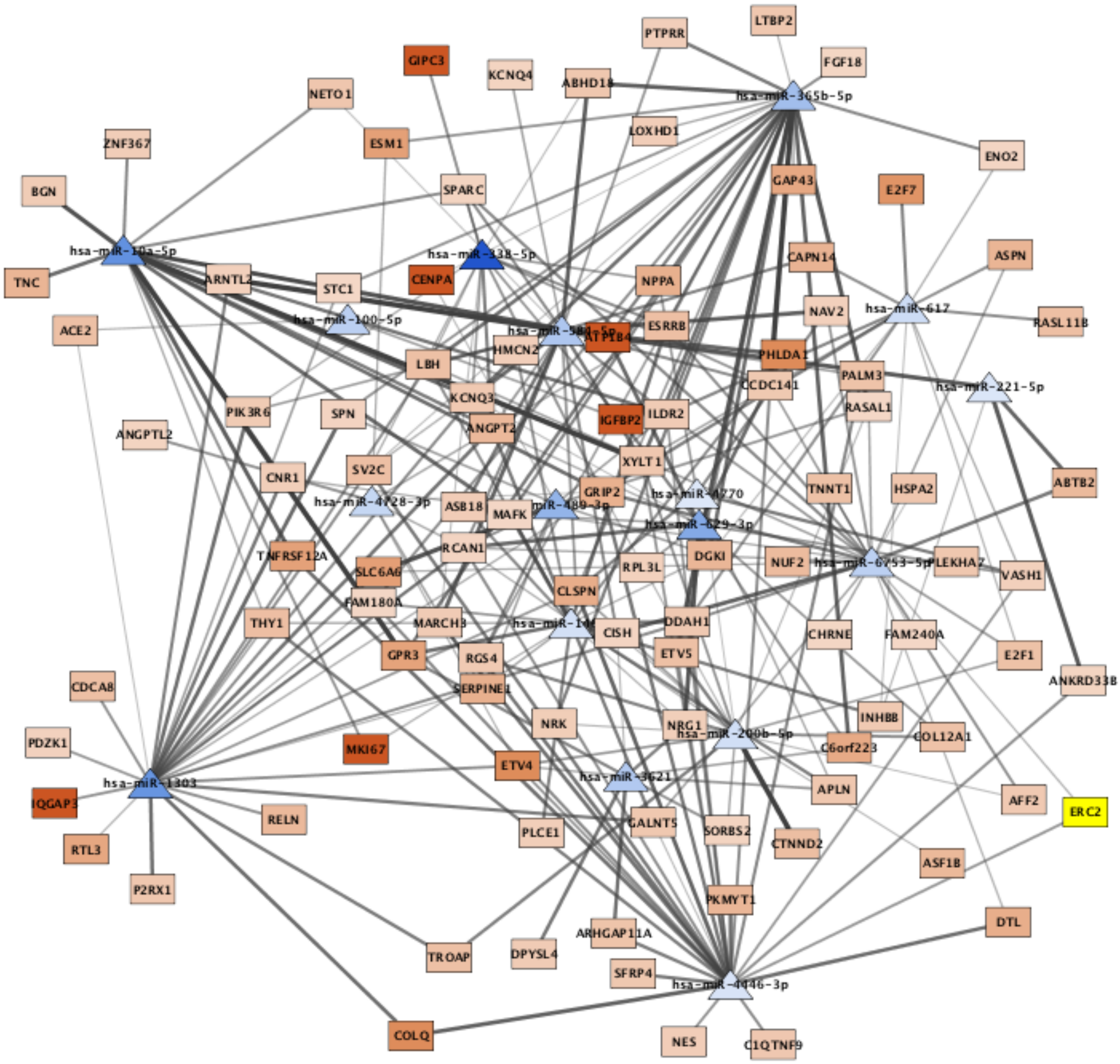

S6 Fig. – miRNA-gene regulatory networks build using Cytoscape. Network includes down-regulated miRNAs and candidate gene targets that were up-regulated in right atrial biopsies of AF patients compared to control subjects. Triangle nodes represent down-regulated miRNAs with blue colour intensity according to FC. Rectangle nodes represent up-regulated genes with red colouring according to FC. Edges connecting miRNA and genes are coloured according to Spearman correlation of expression data. The darker the line, the closer to -1 is the correlation. All miRNA-gene pairs with correlation above -0.5 were excluded from the network. AF – atrial fibrillation; SR – sinus rhythm; FC – fold-change

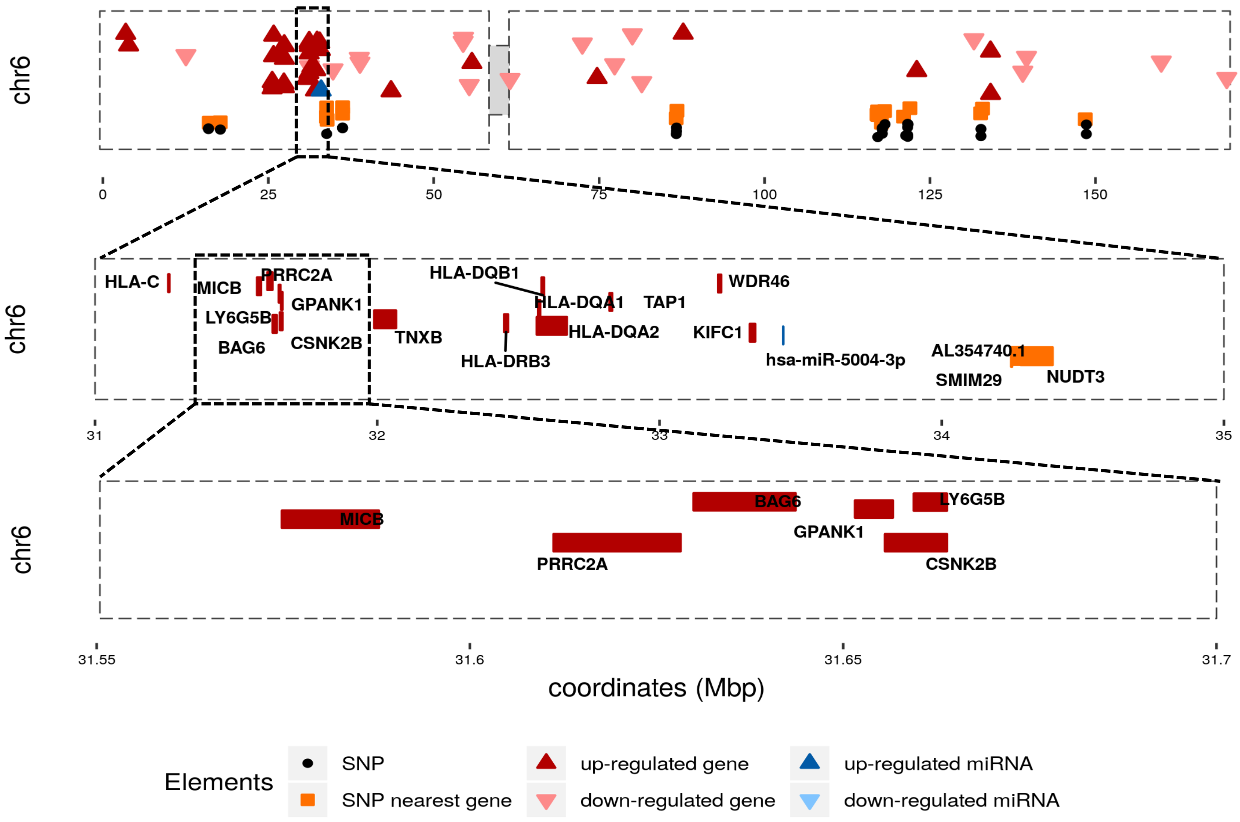

S7 Fig. – Chromosome enrichment of AF related genetic elements, including AF associated SNPs and differentially expresses genes and miRNAs in right atrium biopsies of AF patients compared to sinus rhythm controls. Zoom in on the enrichment cluster 298 identified in the p arm of chromosome 6 including genes *PRRC2A*, *BAG6*, *GPANK1*, *CSNK2B* and *LY6G5B,* all up-regulated in AF patients. AF – atrial fibrillation; SNPs – single nucleotide polymorphisms; Mbp – million base pairs
